# Developmental changes in brain activity of heterozygous *Scn1a* knockout rats

**DOI:** 10.3389/fneur.2023.1125089

**Published:** 2023-03-14

**Authors:** Mayu Tahara, Norimichi Higurashi, Junichi Hata, Masako Nishikawa, Ken Ito, Shinichi Hirose, Takehito Kaneko, Tomoji Mashimo, Tetsushi Sakuma, Takashi Yamamoto, Hirotaka James Okano

**Affiliations:** ^1^Department of Pediatrics, The Jikei University School of Medicine, Minato-ku, Tokyo, Japan; ^2^Division of Regenerative Medicine, The Jikei University School of Medicine, Minato-ku, Tokyo, Japan; ^3^Graduate School of Human Health Sciences, Tokyo Metropolitan University, Arakawa-ku, Tokyo, Japan; ^4^Clinical Research Support Center, The Jikei University School of Medicine, Minato-ku, Tokyo, Japan; ^5^General Medical Research Center, School of Medicine, Fukuoka University, Fukuoka, Japan; ^6^Division of Fundamental and Applied Sciences, Graduate School of Science and Engineering, Iwate University, Morioka, Japan; ^7^Division of Animal Genetics, Laboratory Animal Research Center, Institute of Medical Science, University of Tokyo, Minato-ku, Tokyo, Japan; ^8^Division of Integrated Sciences for Life, Graduate School of Integrated Sciences for Life, Hiroshima University, Hiroshima, Japan

**Keywords:** bumetanide (BTN), developmental and epileptic encephalopathy (DEE), gamma-aminobutyric acid (GABA), functional neuroimaging, Dravet syndrome (DS)

## Abstract

**Introduction:**

Dravet syndrome (DS) is an infantile-onset developmental and epileptic encephalopathy characterized by an age-dependent evolution of drug-resistant seizures and poor developmental outcomes. Functional impairment of gamma-aminobutyric acid (GABA)ergic interneurons due to loss-of-function mutation of *SCN1A* is currently considered the main pathogenesis. In this study, to better understand the age-dependent changes in the pathogenesis of DS, we characterized the activity of different brain regions in *Scn1a* knockout rats at each developmental stage.

**Methods:**

We established an *Scn1a* knockout rat model and examined brain activity from postnatal day (P) 15 to 38 using a manganese-enhanced magnetic resonance imaging technique (MEMRI).

**Results:**

*Scn1a* heterozygous knockout (*Scn*1*a*^+/−^) rats showed a reduced expression of voltage-gated sodium channel alpha subunit 1 protein in the brain and heat-induced seizures. Neural activity was significantly higher in widespread brain regions of *Scn*1*a*^+/−^ rats than in wild-type rats from P19 to P22, but this difference did not persist thereafter. Bumetanide, a Na^+^-K^+^-2Cl^−^ cotransporter 1 inhibitor, mitigated hyperactivity to the wild-type level, although no change was observed in the fourth postnatal week. Bumetanide also increased heat-induced seizure thresholds of *Scn*1*a*^+/−^ rats at P21.

**Conclusions:**

In *Scn*1*a*^+/−^ rats, neural activity in widespread brain regions increased during the third postnatal week, corresponding to approximately 6 months of age in humans, when seizures most commonly develop in DS. In addition to impairment of GABAergic interneurons, the effects of bumetanide suggest a possible contribution of immature type A gamma-aminobutyric acid receptor signaling to transient hyperactivity and seizure susceptibility during the early stage of DS. This hypothesis should be addressed in the future. MEMRI is a potential technique for visualizing changes in basal brain activity in developmental and epileptic encephalopathies.

## 1. Introduction

Dravet syndrome (DS) is an infantile-onset developmental and epileptic encephalopathy characterized by pharmacoresistant seizures, seizure susceptibility at high temperatures, cognitive and behavioral impairments after the first year of life, and motor disorders including ataxia and crouch gait (1–3). High premature mortality (4–20%) has been reported, and the main causes of death include sudden unexpected death, *status epilepticus*, and acute encephalopathy (3, 4).

DS is characterized by a cluster of age-related electroclinical features.

Unilateral/generalized convulsive *status epilepticus* (CSE) frequently occurs in the early stage of the disease. Thereafter, multiple seizure types, including focal, myoclonic, absence, and atonic seizures, appear. At later stages, interictal electroencephalography (EEG) shows diffuse and/or focal epileptic discharges, which are usually not observed during infancy (5). After 5 years of age, the occurrence of CSE and other seizure types decreases, whereas brief nocturnal generalized tonic-clonic seizures exhibit life-long occurrence (2, 3).

Heterozygous loss-of-function mutations in the *SCN1A* gene have been identified in over 80% of patients with DS (2, 6). *SCN1A* encodes voltage-gated sodium channel alpha subunit 1 (Na_V_1.1) and is predominantly expressed in the parvalbumin-positive gamma-aminobutyric acid (GABA)ergic interneurons (7, 8). These interneurons are the main source of phasic inhibition in the brain, and hence, impaired cerebral inhibition due to Na_V_1.1 haploinsufficiency in these neurons is considered the major mechanism underlying DS epilepsy (7, 9, 10). However, to establish better treatment strategies and improve the disease outcome, it is essential to comprehensively understand the pathogenesis of the age-dependent phenotypic changes that occur in DS.

To address this issue, we employed manganese-enhanced magnetic resonance imaging (MEMRI) in newly established *Scn1a* knockout rats, a technique used to visualize *in vivo* brain activity by using properties of manganese ions (Mn^2+^). Mn^2+^ ions shorten the T1 relaxation time in tissues where they accumulate and act as a calcium ions analog in neuronal tissue. They could also enter the firing neurons via voltage-gated calcium channels, and get transported to the adjacent neurons (11). Accordingly, Mn^2+^ accumulate in areas with higher neuronal activity, resulting in hyperintensity on T1-weighted images. Therefore, MEMRI has been widely applied as an *in vivo* method to understand the brain activity in various neurological diseases (12). The kinetic properties of Mn^2+^, which are slowly distributed in the brain over a 24-h period after systemic administration (11, 13, 14) suggest that MEMRI is more suitable for evaluating basal brain activity than for transient epileptic activity like seizures. Previous reports have also reported difficulty in detecting brain activity in *status epilepticus* using MEMRI (15–17), suggesting the usefulness of MEMRI as a tool for detecting developmental changes in the brain activity of different regions in epilepsy and analyzing the underlying pathogenesis.

Here, we evaluated the age-dependent alteration in the brain activity of different regions in the *Scn1a* knockout rats using MEMRI. Previous studies on the pathogenesis of DS have mainly used mouse models of *Scn1a* knockout (18–20). We used rats because they have larger brains than mice, allowing for more accurate evaluation of brain activity by MRI, even in pups. In addition, when using mice, longer imaging times are required to perform high-resolution MRI experiments, and there are concerns about the effects of prolonged anesthesia and the imaging environment on the images. Coil fitting is also an important factor affecting the results of the analysis when targeting small brains. Furthermore, as compared to mice, the developmental patterns in rats, including developmental changes in the Na^+^-K^+^-2Cl^−^ cotransporter 1 (NKCC1) and K^+^-Cl^−^ cotransporter 2 (KCC2), have been reported to have more similar to those in humans (21).

Although we established the rat model for DS, the phenotype was milder than that of *Scn1a* knockout mice, in terms of lower frequency of spontaneous seizures and mortality rate. However, we hypothesized these rat models may be better suited to evaluate the pathogenic alterations directly caused by *Scn1a* defects, while minimizing the influences of spontaneous seizures and malnutrition.

## 2. Materials and methods

### 2.1. Transcription activator-like effector nuclease -mediated genome editing in rats

A pair of TALENs targeting exon 1 of rat *Scn1a* (Ensembl: ENSRNOG00000053122) was designed and constructed using a two-step assembly method with a Platinum Gate kit as previously reported (22). Assembled sequence was 5′-TGCAGGATGACAAGATGgagcaaacagtgcttGTACCACCAGGACCTGA-3′, where uppercase and lowercase letters indicate TALEN target sequences and spacer sequence, respectively. TALENs were microinjected into fertilized eggs of Fisher 344 (F344) rats and transferred into the oviducts of pseudopregnant female Wistar rats, as previously described (23). Genomic DNA was extracted from the tail using the GENEXTRACTOR TA-100 automatic DNA purification system (Takara Bio) and amplified with specific primer sets (forward 5′-TCCTCACTTGTTGGGTCTCA-3′, reverse 5′-TCAGGGTGACTTCAGCATTTC-3′). The polymerase chain reaction (PCR) products were directly sequenced using the BigDye terminator v3.1 cycle sequencing mix and the standard protocol for an Applied Biosystems 3130 DNA Sequencer (Life Technologies). Rats from the fifth generation or later were used in all experiments. Genotypes were assessed by running the PCR products from ear DNA on a Caliper electrophoresis system at postnatal day (P9). Only male rats were used in experiments to eliminate sex differences.

### 2.2. F344-*Scn1a*^em1kyo^ rats

This study was approved by the Animal Research Committee of Kyoto University and the Institutional Animal Care and Use Committee of the Jikei University School of Medicine (protocol number: 2015-125C7). All procedures were conducted according to the Guidelines of the Proper Conduct of Animal Experiments of the Science Council of Japan (2006). The principles outlined in the ARRIVE guidelines, including the 3R concept, were followed when planning the experiments. P15 to 38 rats were used in this study (*n* =162, body weight range 21–124 g). Rats were weaned on P22 and housed in polycarbonate cages under temperature-controlled conditions (temperature: 24–25°C; relative humidity: 50–60%) with a 12/12-h light–dark cycle. They had free access to water and pelleted food (CE-2, CLEA Japan, Inc.).

### 2.3. Western blot analysis

Protein extraction from rat brains was performed at P13 using an ULTRARIPA kit for lipid rafts (F015; BioDynamics Laboratory Inc.); the reagent solution contained 50 mM Tris-HCl (pH 8.0), 150 mM NaCl, 1% NP-40 alternative, 0.1% SDS, and 0.5% sodium deoxycholate. Proteins were separated using SDS-PAGE and transferred to polyvinylidene difluoride membranes (Millipore). The membranes were blocked with 5% nonfat skim milk overnight at 4°C and incubated in primary antibody solution containing rabbit anti- voltage-gated sodium channel alpha subunit 1 (Na_V_1.1) (ASC-001, 1:200; Alomone Labs) and mouse anti-β-actin (A1978, 1:1000; Sigma-Aldrich) for 1 h at room temperature and sequentially incubated with secondary antibodies containing anti-Rb IgG HRP (AP307P, 1:2000; Millipore) and anti-mouse IgG HRP (AP308P, 1:2000; Millipore) for 1 h at room temperature. The signal was detected using Chemiluminescence HRP Substrate (Takara Bio) on the LAS3000 Mini apparatus (FUJI FILM). Semiquantitative analysis of the signal was performed using ImageJ software (National Institute of Health).

### 2.4. EEG recordings

All surgical procedures were performed under isoflurane anesthesia (4–1.5%) at P14. Epidural screw electrodes for EEG were mounted on the skull bilaterally over the somatosensory cortex (2.0 mm lateral to midline, 2.0 mm posterior to bregma) and the cerebellum (1.0 mm lateral to midline, 2.0 mm posterior to lambda) as reference electrodes. Electromyography (EMG) electrodes were inserted into the suprascapular area. Heat-induced seizures were triggered in rats as described below, and behavioral seizures, video–EEG, and EMG were recorded using a PowerLab system (AD Instruments) at P21. Data analysis was performed with LabChart software (AD Instruments).

### 2.5. Animal preparation for MRI

Rats were initially anesthetized with 3% isoflurane in 30/70% O_2_/N_2_ in a closed chamber. Rats were laid in the prone position on a dedicated animal bed heated with warm circulating water. Anesthesia with 1.5–2.5% isoflurane in 30/70% O_2_/N_2_ was delivered to the spontaneously breathing animals through a snout mask, and the respiratory rate was maintained at 50–60 breaths/min. The rectal temperature was carefully monitored and maintained at 36.5 ± 0.5°C by circulating warm water under the bed.

### 2.6. MRI measurements and image analysis

Imaging was performed using a 9.4 T MRI 94/20 system (Bruker BioSpin GmbH) with a mouse head surface coil and a 72-mm transmit coil. We acquired T1 maps for the MRI study. The scanning parameters were as follows: T1WI for T1 map: echo time, 6.5 ms; repetition times, 525, 1050, 2100, 4200, and 8400 ms; averages, 2; scan time, 1 h 3 min 28 s 350 ms; rapid acquisition with relaxation enhancement factor, 1; slices, 24; slice thickness, 0.8 mm; image matrix, 140 × 140; field of view, 16.0 × 16.0 mm; resolution, 0.114 × 0.114 mm; and slice gap, 0.2 mm. T1 value for each ROI was calculated for each T1 map using ImageJ software (National Institutes of Health).

### 2.7. MEMRI

An isotonic solution of MnCl_2_·4H_2_O (203734; Sigma-Aldrich) was prepared at a concentration of 100 mM in 100 mM bicine solution, as previously described (24). Then, the MnCl_2_ solution was diluted to 50 mM with saline, and the pH was adjusted to 7.4.

We intraperitoneally administered a 50 mM solution of MnCl_2_ immediately after the initial MRI acquisition, twice at 1-h intervals, to get a total dose of 66 mg/kg. MEMRI acquisition was performed 24 h after the first administration of MnCl_2_ based on the kinetics of intracerebral Mn^2+^ concentration (11). The imaging area was adjusted to the same position as in the initial MRI to facilitate comparison of T1 values at the same ROI.

### 2.8. Systemic bumetanide or saline administration

The effective systemic dose of bumetanide on neonatal seizures in rats is 0.1–0.5 mg/kg (21, 25). We administered 0.2 mg/kg of bumetanide twice a day in repeated doses as previously described (26). Bumetanide (B3023; Sigma-Aldrich) was dissolved in 100% ethyl alcohol and then diluted with 0.9% saline to a final concentration of 0.05 mg/mL. Bumetanide solution (0.2 mg/kg) or equal volumes of 0.9% saline (4 mL/kg) was injected twice daily from P12 to P20 or P26. MEMRI was performed on P21 or P27 for each rat.

### 2.9. Induction of heat-induced seizures

*Scn*1*a*^+/^− rats were subjected to heat-induced seizures on P21 or P27 after daily administration of bumetanide or 0.9% saline from P12 to P20 or P26. Hyperthermia was induced by placing the rats in a bath filled 10 cm deep with 45°C water, as previously described (27), for a maximum of 5 min or until a seizure occurred. When a seizure occurred, the rats were removed immediately from the bath and monitored until recovery. For each rat, we recorded the latency from water contact to seizure onset, the seizure duration, and a score based on the most severe seizure observed. Seizure severity was scored based on Racine stages (28): 0, no response; 1, oral and facial movement; 2, head nodding; 3, forelimb clonus; 4, forelimb clonus with rearing; and 5, generalized tonic-clonic seizures and falling. All procedures were recorded using a video camera. We did not measure body temperature because it was difficult and would be inaccurate to measure body temperature in a water bath.

### 2.10. Statistical analyses

Statistical analysis was performed using SAS 9.4 software (SAS Institute Inc.) and GraphPad Prism 8 software (GraphPad Software Inc.). We used ANCOVA to compare T1_post_ (T1 values resulting from MEMRI T1 mapping) and adjusted for native T1 values (T1_pre_), applying a split-split-plot design under different genotypes, developmental stages, and regions (29). Genotypes and developmental stages were applied to whole plots (animals), and regions were applied to split-split-plots (regions in an animal) in the MEMRI experiment at each stage of development. In the MEMRI experiment with and without bumetanide, genotype and drug (bumetanide or saline) were applied to whole plots (animals), and regions were applied to split-split-plots (regions in an animal). The replicate effect was combined into whole-plot error, including Error(A) and Error(B) (29), and Type III Sum of Squares in SAS was used as the main result. The Scheffé or Tukey multiple-comparison test was performed with split-split-plot error to compare genotypes in each region at each developmental stage or to compare bumetanide and normal saline at each region in each genotype. Differences in seizure phenotypes were assessed using the Mann–Whitney *U*-test. The *p*-values were two-sided, and *p* < 0.05 was considered statistically significant.

## 3. Results

### 3.1. Generation of *Scn1a* knockout rats

**Figure 1:**
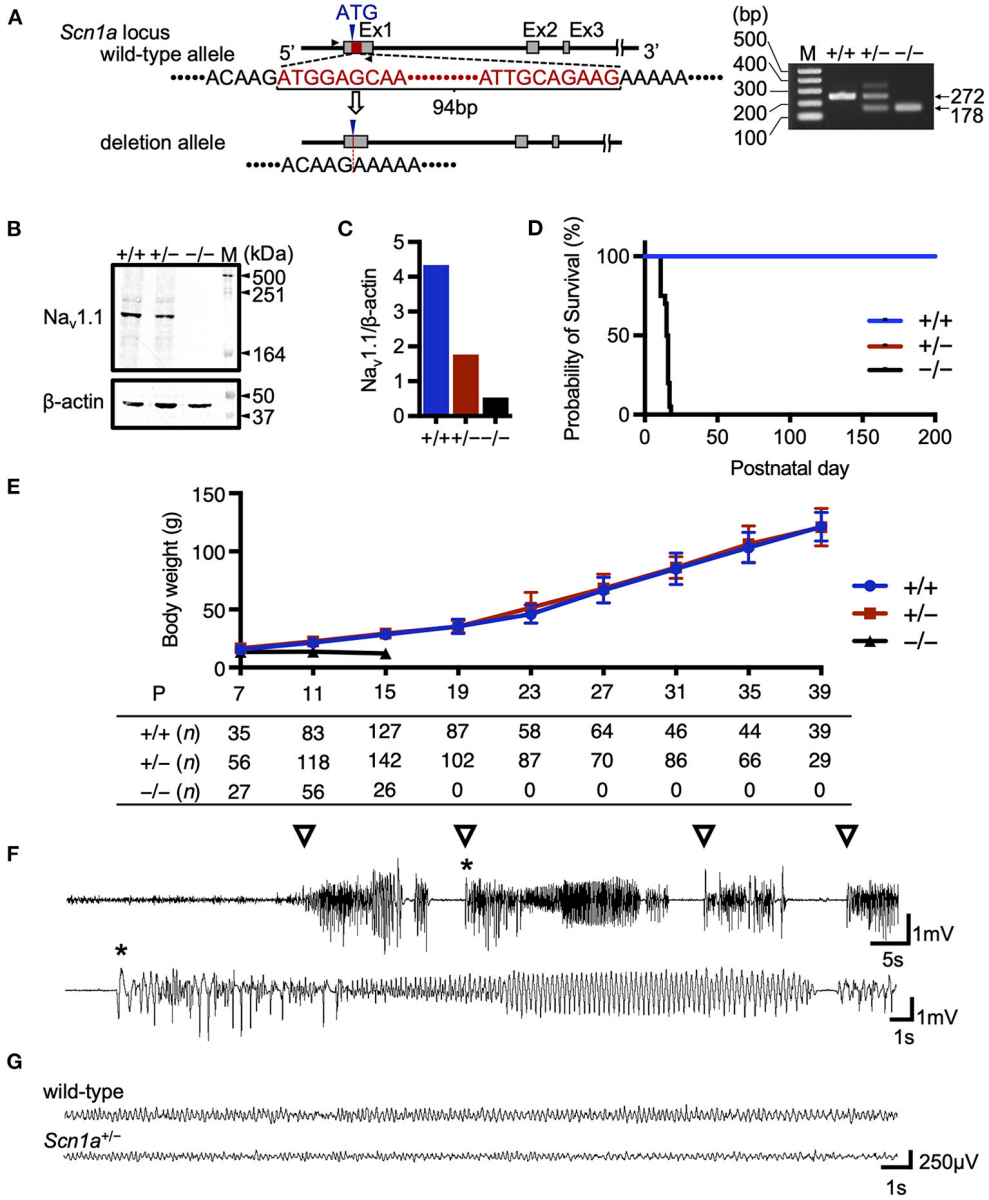
Targeting construct and generation of global *Scn1a* knockout rats. **(A)** The arrows show the position of the genotyping primer set (forward 5′-TAATAACTTTTAATGCTATC-3′, reverse 5′-CTTCCCAGCTTCCAAGTCAC-3′). Genotype analysis shows a 272 bp band in the wild-type allele and a 178 bp band in the *Scn*1*a*^em1kyo^ mutant. The upper band of the *Scn*1*a*^em1kyo^ mutant is nonspecific. Genotypes are indicated by +/+ for wild-type, +/– for heterozygous, and –/– for homozygous animals. **(B)** Western blotting of brain membrane proteins from wild-type (+/+), heterozygous (+/–), and homozygous (–/–) *Scn1a* rats at P13 using an anti- voltage-gated sodium channel alpha subunit 1 (Na_V_1.1) antibody. β-actin was used as the internal control. Original blots/gels are presented in Supplementary Figure 1. **(C)** Relative Na_V_1.1 protein levels normalized to β-actin. **(D)** Survival curves of wild-type (*n* = 15), *Scn*1*a*^+^/− (*n* =15), and *Scn*1*a*^−/−^ (*n* = 20) rats. *Scn*1*a*^−/−^ rats exhibited ataxia and seizures from postnatal day (P) 10, gradually progressing to weight loss and complete loss of postural control. They became inactive and did not survive beyond P18. In wild-type and *Scn*1*a*^+^/− rats, there were no spontaneous deaths. **(E)** Body weight curves of wild-type (blue circle), *Scn*1*a*^+^/− (red square), and *Scn*1*a*^−/−^ (black triangle) rats. The sample size is shown below the graph. *Scn*1*a*^+^/− rats showed no difference in body weight compared to wild-type rats. Data are presented as mean ± standard deviation. **(F)** Ictal electroencephalography (EEG) recording of heat-induced seizures from *Scn*1*a*^+^/− rats at P21. Spiking activity was recorded, and generalized tonic-clonic seizures were observed. Open triangle indicates seizure onset. Asterisk indicates the expanded EEG trace of spiking activity. **(G)** Representative interictal EEG recordings from wild-type and *Scn*1*a*^+^/− rats at P21. No interictal epileptic discharge was found.

In this study, we developed a global *Scn1a* knockout rat model using the TALEN-mediated genome editing technique by generating a frameshift in exon 1 of the *Scn1a* gene (Figure 1A). TALEN mRNAs targeting *Scn1a* exon 1 were microinjected into fertilized eggs of F344 rats, and a 94-base-pair deletion in exon 1 was identified in a resulting founder mutant rat (F344-*Scn1a*^*em*1*Kyo*^). Reduced Na_V_1.1 protein expression was confirmed in the brains of heterozygous (*Scn*1*a*^+/^−) and homozygous (*Scn*1*a*^−*/*−^) knockout rat offspring (Figures 1B, C and Supplementary Figure 1).

### 3.2. *Scn1a* knockout rats exhibited key phenotypic features of *Scn1a*-related epilepsies, but were milder than those of DS

As previously reported in *Scn1a* knockout mice (9, 18–20), *Scn*1*a*^−/−^ rats died at approximately the second postnatal week, but no spontaneous deaths were observed in *Scn*1*a*^+/−^ rats until at least P200 (Figure 1D). The mean body weight of *Scn*1*a*^+/−^ rats was not significantly different from that of wild-type rats during the experimental period (Figure 1E). Heat-induced seizures were observed in all investigated *Scn*1*a*^+/−^ rats (*n* = 26) at 45°C water, a temperature that did not induce seizures in wild-type rats (Figure 1F, Supplementary Video 1). Up to the third postnatal week, spontaneous convulsive seizures were rarely observed and EEG recordings showed no interictal epileptiform discharges or ictal activities during recording in the *Scn*1*a*^+/−^ rats (Figure 1G). Notably, in older *Scn*1*a*^+/−^ rats, some seizures were provoked by acoustic or vibrational stimuli. These findings indicate that *Scn*1*a*^+/−^ rats have milder symptoms than those reported in *Scn*1*a*^+/−^ mice, suggesting their phenotype may be closer to the genetic epilepsy with febrile seizures plus phenotype.

### 3.3. MEMRI revealed increased neural activity in widespread brain regions of *Scn*1*a*^+/−^ rats from P19 to P22

**Figure 2:**
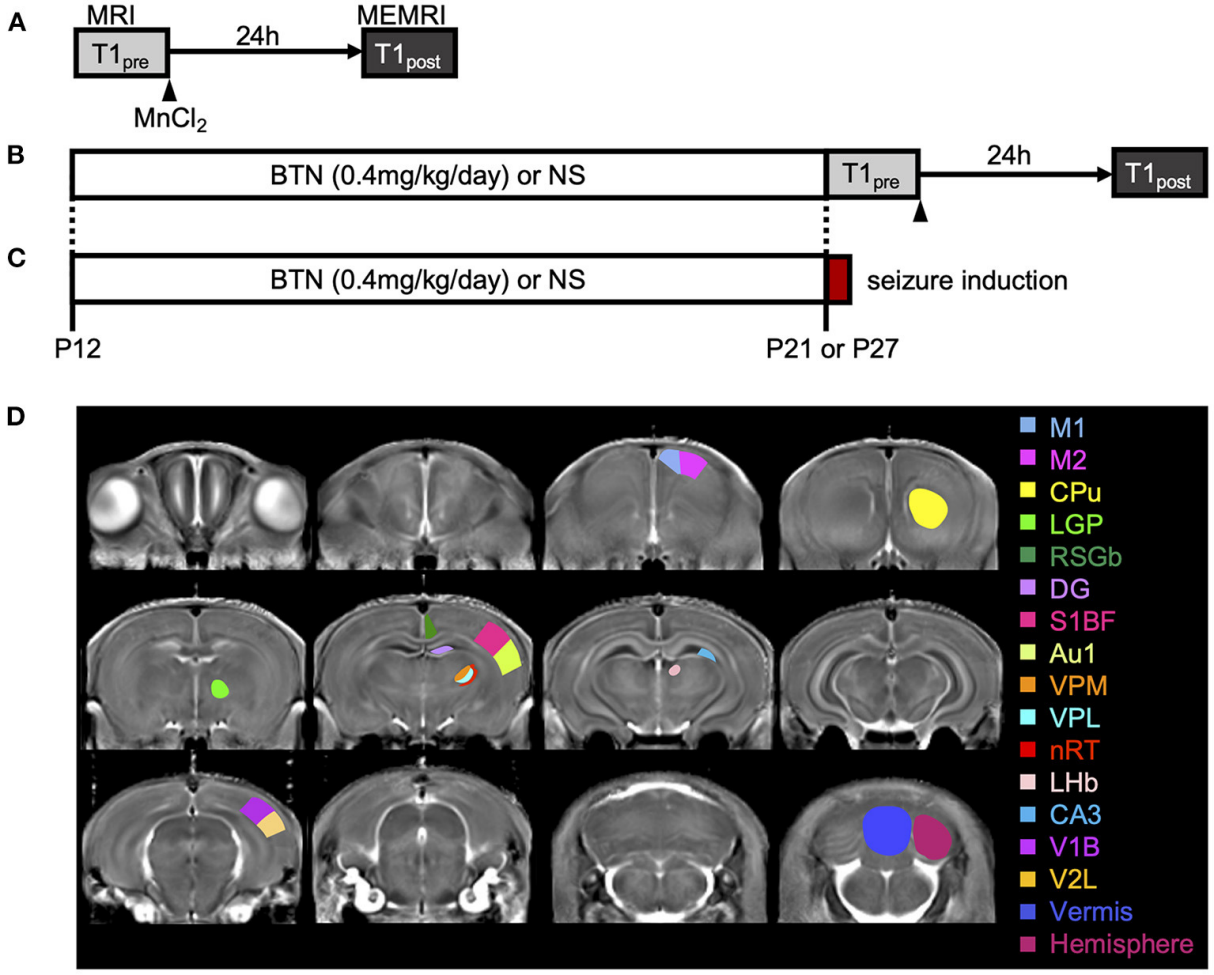
Schematic diagram of the experiments. **(A)** Manganese-enhanced magnetic resonance imaging (MEMRI) experiment. Manganese chloride was intraperitoneally administered immediately after the first T1 mapping, which was reconfirmed 24 h later. **(B)** MEMRI experiment in rats treated with bumetanide (BTN) or normal saline (NS). BTN solution (0.2 mg/kg) or an equal volume of NS (4 mL/kg) was injected intraperitoneally twice a day beginning at postnatal day (P) 12, and MEMRI was performed for each rat on P21 or P27. **(C)** Heat-induced seizure experiment in rats treated with BTN or NS. After daily injections of BTN or NS, seizures were induced in rats by a warm bath at P21 or P27. **(D)** Representative coronal T1 map and regions of interest. Regions were manually obtained using the Paxinos and Watson atlas. The image shows the average of T1 maps. Colors indicate 17 different brain regions, which are overlaid on averaged images of the MEMRI T1 map. M1, primary motor cortex; M2, secondary motor cortex; CPu, caudate-putamen (striatum); LGP, lateral globus pallidus; RSGb, retrosplenial granular b cortex; DG, dentate gyrus; S1BF, primary somatosensory cortex, barrel field; Au1, primary auditory cortex; VPM, ventral posteromedial thalamic nucleus; VPL, ventral posterolateral thalamic nucleus; nRT, reticular thalamic nucleus; LHb, lateral habenular nucleus; CA3, CA3 field of the hippocampus; V1B, primary visual cortex, binocular area; V2L, secondary visual cortex, lateral area; Vermis, cerebellar vermis; Hemisphere, cerebellar hemisphere.

**Figure 3:**
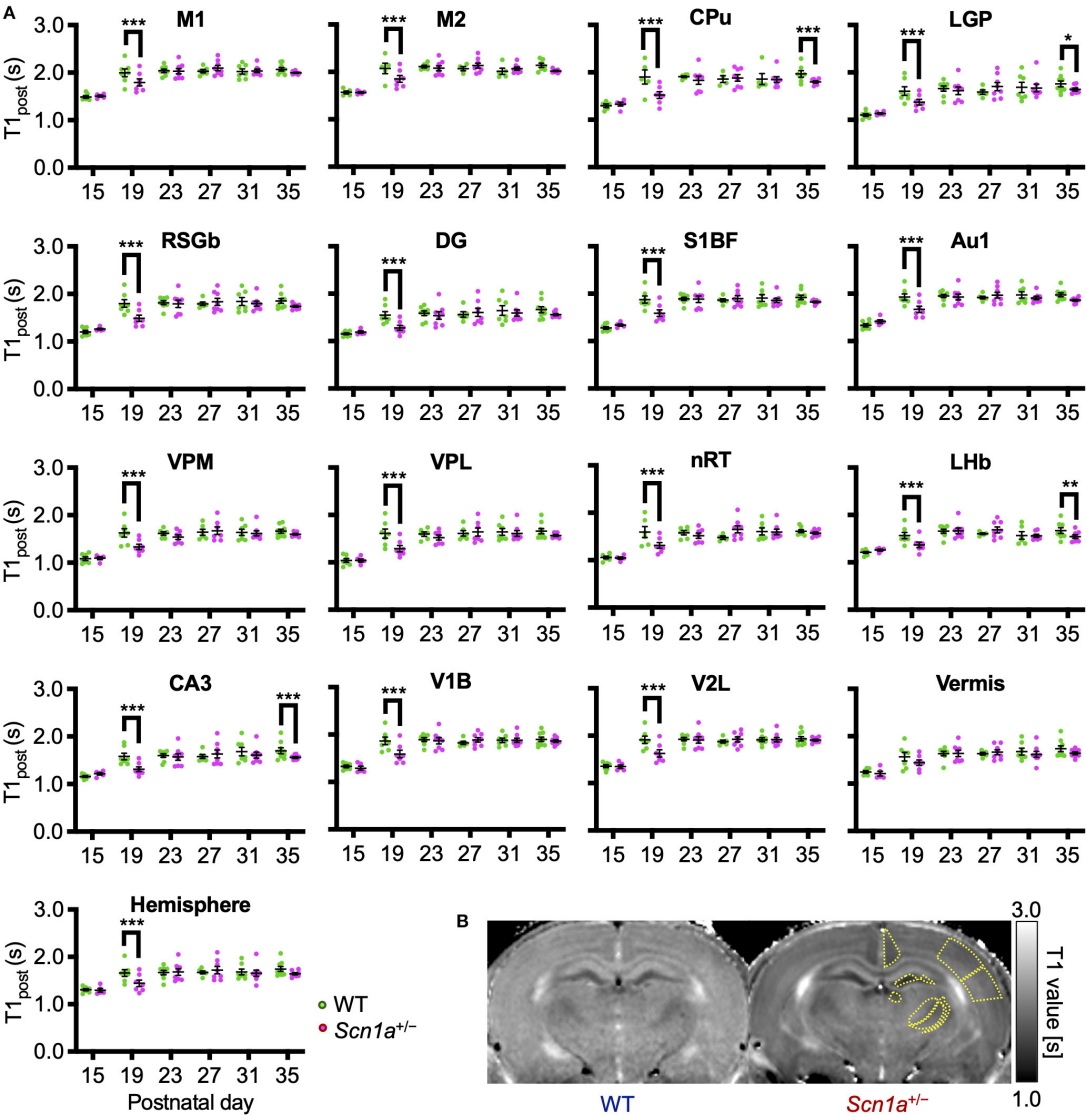
Age-dependent changes in T1_post_ in each brain region of wild-type and *Scn*1*a*^+/−^ rats. **(A)** T1_post_ showed age-dependent changes in each region of wild-type (WT, green circle) and *Scn*1*a*^+/−^ rats (*Scn*1*a*^+/−^, magenta circle). Tukey multiple-comparison test showed a significant decrease in T1_post_ of *Scn*1*a*^+/−^ rats during P19–22: M1(mean difference 0.205s; 95% CI 0.073–0.337s), M2 (0.198s; 0.066–0.330s), CPu (0.355s;−0.203–0.467s), LGP (0.247s; 0.115–0.380s), RSGb (0.318s; 0.203–0.467s), DG (-0.272s; 0.140–0.404s), S1BF (0.299s; 0.167–0.431s), Au1 (0.269s; 0.137–0.401s), VPM (0.286s; 0.154–0.419s), VPL (0.316s; 0.184–0.448s), nRT (0.280s; 0.142–0.418s), LHb (0.202s; 0.070–0.334s), CA3 (0.277s; 0.145–0.409s), V1B (0.278s; 0.147–0.410s), V2L (0.291s; 0.159–0.423s), Vermis (0.126s;−0.006–0.258s), and Hemisphere (0.229s; 0.097–0.361s). These differences disappeared after P23, but are significantly decreased during P35–38: CPu (0.148s; 0.028–0.268s), LGP (0.120s; 0.000–0.240s), LHb (0.136s; 0.012–0.260s), and CA3 (0.143s; 0.023–0.263s). Tukey's multiple-comparison test; *p < 0.05, ** *p* < 0.01, *** *p* < 0.001. Data are presented as mean ± standard error of the mean (SEM). Dots represent individual data points. **(B)** Representative MEMRI T1 map at P21. Yellow lines show part of regions of interest. MEMRI, manganese-enhanced magnetic resonance imaging; M1, primary motor cortex; CI, confidence interval; M2, secondary motor cortex; CPu, caudate-putamen (striatum); LGP, lateral globus pallidus; RSGb, retrosplenial granular b cortex; DG, dentate gyrus; S1BF, primary somatosensory cortex, barrel field; Au1, primary auditory cortex; VPM, ventral posteromedial thalamic nucleus; VPL, ventral posterolateral thalamic nucleus; nRT, reticular thalamic nucleus; LHb, lateral habenular nucleus; CA3, CA3 field of the hippocampus; V1B, primary visual cortex, binocular area; V2L, secondary visual cortex, lateral area; Vermis, cerebellar vermis; Hemisphere, cerebellar hemisphere; P, postnatal day.

To identify disease-specific alterations in regional brain activities of developing *Scn*1*a*^+/−^ rats, we employed T1 mapping in a MEMRI experiment. This technique represents the T1 relaxation time as the T1 value for each pixel; decrease in the T1 value in MEMRI indicates increase in neural activity. Several MEMRI studies have used T1 mapping to quantitatively compare brain activity as T1 value or R1(1/T1) and successfully identified regions that show disease-specific changes in brain activity (30, 31). Therefore, we applied this technique to quantitatively compare the regional activity of wild-type and *Scn*1*a*^+/−^ rats. As T1 values might be influenced by myelination (32, 33), we first investigated T1_pre_ before performing MEMRI in wild-type (*n* = 44, body weight range 21–118 g) and *Scn*1*a*^+/−^ rats (*n* = 44, 25–124 g) from P15 to P38 (Supplementary Figure 2). MnCl_2_ was intraperitoneally administered immediately afterwards, and MEMRI T1 mapping was performed on the same rats to investigate T1_post_ (Figure 2A). We compared T1_post_ values, adjusted for T1_pre_, under different genotypes, developmental stages, and regions. Region of interests (ROIs) were defined in 17 areas in the cortex, subcortex, and cerebellum (Figure 2D). The developmental stages of rats were defined according to a previous report (34): P15–18 (neonatal period), P19–22 (infancy), P23–26 (weaning period), P27–30 (early juvenile period), P31–34 (juvenile period), and P35–38 (prepuberty). If T1_post_ or T1_pre_ could not be measured in a region, both values were excluded from the analysis. The sample size for each postnatal day and each ROI is shown in Table 1. Analysis of covariance (ANCOVA) for T1_post_ showed neither a significant main effect of genotype [F_(1, 76)_ = 3.19, *p* = 0.08] nor an interaction among the developmental stages, genotypes, and ROIs [F_(80, 1194)_ = 1.28, *p* = 0.05]. In *Scn*1*a*^+/−^ rats, however, a significant decrease in T1_post_ was observed compared to wild-type rats at P19–22 in all ROIs, except the cerebellar vermis, by the Tukey multiple-comparison test with respect to the interaction (*p* < 0.001; cerebellar vermis, *p* = 0.11), while the decrease was not significant at P15–18 or P23–34 (Figures 3A, B). At P35–38, a significant decrease reappeared in the hippocampal CA3, lateral habenular nucleus, and basal ganglia (CA3, *p* < 0.001; lateral habenular nucleus, *p* = 0.007; globus pallidus, *p* = 0.05; caudate-putamen, *p* < 0.001). These results demonstrate that neural activity across widespread brain regions is significantly increased in *Scn*1*a*^+/−^ rats at P19–22, which corresponds to the peak onset age of DS in humans (1, 4, 35).

**Table 1:** Sample size for each postnatal day and each region of interest.

**(A)**								
**P**	**Number of rats**		**M1**		**M2**		**CPu**	
	+**/**+	*+**/*** **–**	+**/**+	*+**/*** **–**	+**/**+	*+**/*** **–**	+**/**+	*+**/*** **–**
15–18	7	5	7	5	7	5	7	5
19–22	7	7	7	7	7	7	7	7
23–26	8	8	8	8	8	8	8	8
27–30	6	8	6	8	6	8	6	8
31–34	7	8	7	8	7	8	7	8
35–38	9	8	9	8	9	8	9	8
Total	44	44	44	44	44	44	44	44
**(B)**								
**P**	**LGP**		**RSGb**		**DG**		**S1BF**	
	+**/**+	*+**/*** **–**	+**/**+	*+**/*** **–**	+**/**+	*+**/*** **–**	+**/**+	*+**/*** **–**
15–18	7	5	7	5	7	5	7	5
19–22	7	7	7	7	7	7	7	7
23–26	8	8	8	8	8	8	8	8
27–30	5 (1)	8	6	8	6	8	6	8
31–34	7	8	7	8	7	8	7	8
35–38	9	8	9	8	9	8	9	8
Total	43	44	44	44	44	44	44	44
**(C)**								
**P**	**Au1**		**VPM**		**VPL**		**nRT**	
	+**/**+	*+**/*** **–**	+**/**+	*+**/*** **–**	+**/**+	*+**/*** **–**	+**/**+	*+**/*** **–**
15–18	7	5	7	5	7	5	6 (1)	5
19–22	7	7	7	7	7	7	6 (1)	7
23–26	8	8	6 (2)	7 (1)	6 (2)	7 (1)	6 (2)	7 (1)
27–30	6	8	6	7 (1)	6	7 (1)	4 (2)	8
31–34	7	8	7	8	7	8	7	8
35–38	9	8	9	8	9	8	8 (1)	8
Total	44	44	42	42	42	42	37	43
**(D)**								
**P**	**LHb**		**CA3**		**V1B**		**V2L**	
	+**/**+	*+**/*** **–**	+**/**+	*+**/*** **–**	+**/**+	*+**/*** **–**	+**/**+	*+**/*** **–**
15–18	7	5	7	5	7	5	7	5
19–22	7	7	7	7	7	7	7	7
23–26	8	8	8	8	8	8	8	8
27–30	5 (1)	8	6	8	6	8	6	8
31–34	6 (1)	7 (1)	7	8	7	8	7	8
35–38	8 (1)	8	9	8	9	8	9	8
Total	41	43	44	44	44	44	44	44
**(E)**								
**P**	**Vermis**		**Hemisphere**					
	+**/**+	*+**/*** **–**	+**/**+	*+**/*** **–**				
15–18	7	5	7	5				
19–22	7	7	7	7				
23–26	8	8	8	8				
27–30	6	8	6	8				
31–34	7	8	7	8				
35–38	9	8	9	8				
Total	44	44	44	44				

The numbers in parentheses indicate the rats that could not be measured. +/+ = wild-type; +/– = Scn1a^+/−^ rats. Au1, primary auditory cortex; CA3, CA3 field of the hippocampus; CPu, caudate-putamen (striatum); DG, DG, dentate gyrus; Hemisphere, cerebellar hemisphere; LGP, lateral globus pallidus; LHb, lateral habenular nucleus; M1, primary motor cortex; M2, secondary motor cortex; nRT, reticular thalamic nucleus; P, postnatal days; RSGb, retrosplenial granular b cortex; S1BF, primary somatosensory cortex, barrel field; VPM, ventral posteromedial thalamic nucleus; VPL, ventral posterolateral thalamic nucleus; V1B, primary visual cortex, binocular area; V2L, secondary visual cortex, lateral area; Vermis, cerebellar vermis.

### 3.4. NKCC1 inhibitor rescued the increased brain activity in *Scn*1*a*^+/^− rats at P21

**Figure 4:**
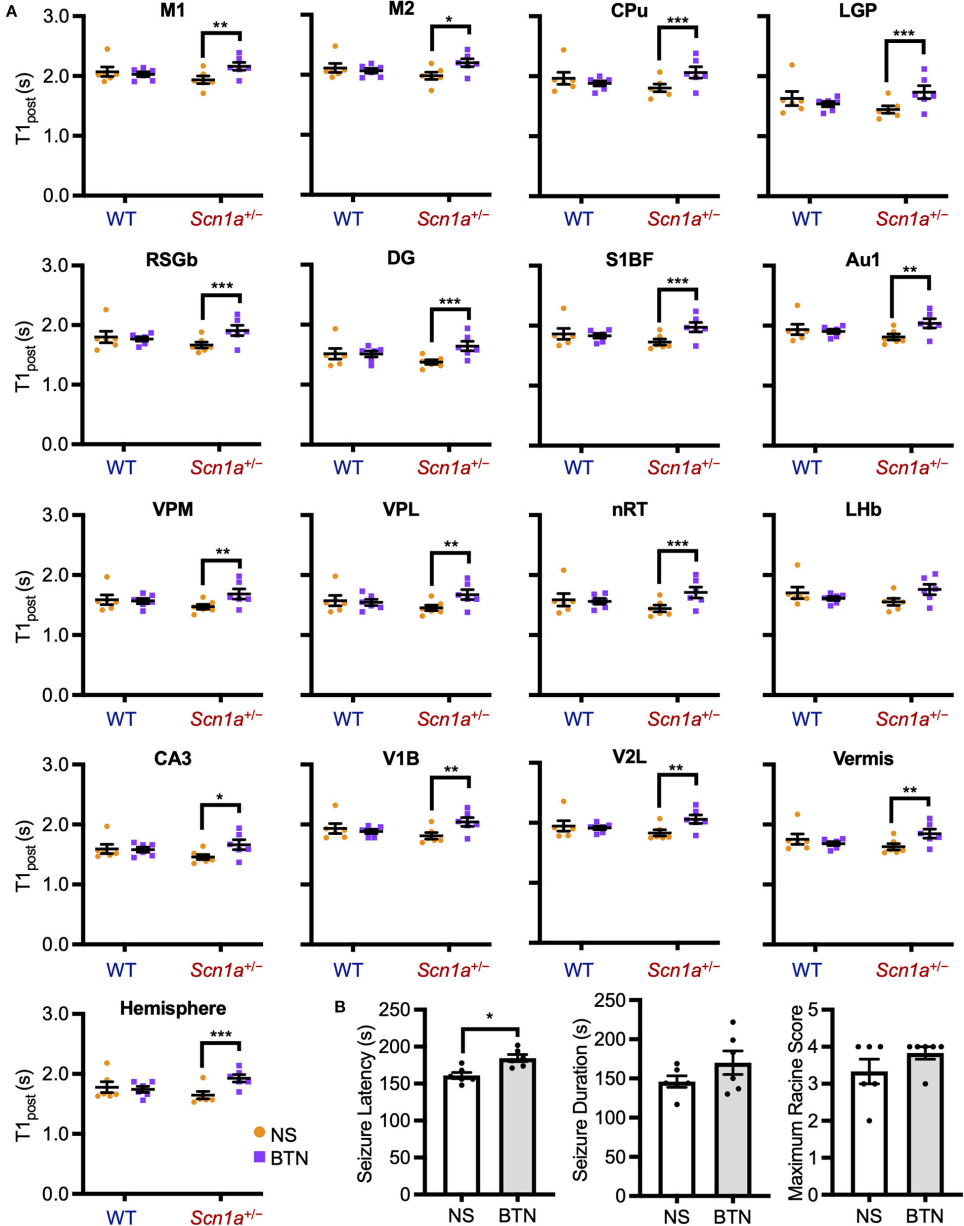
T1_post_ and seizure characteristics after administration of BTN or NS. **(A)** Scheffé multiple-comparison test of T1_post_ in rats at P21 after the administration of BTN (orange circle) or NS (purple square). *Scn*1*a*^+/−^ rats showed a significant increase in the T1_post_ of widespread brain regions in the BTN-treated group compared to the NS-treated group: M1 (mean difference−0.225s; 95% CI−0.423-−0.027s), M2 (-0.210s;−0.408−0.011s), CPu (-0.260s;−0.458−0.062s), LGP (-0.288s;−0.486−0.090s), RSGb (-0.236s;−0.435−0.037s), DG (-0.265s;−0.463−0.067s), S1BF (-0.235s;−0.435−0.035s), Au1 (-0.222s;−0.421−0.024s), VPM (-0.216s;−0.414−0.018s), VPL (-0.223s;−0.420−0.025s), nRT (-0.272s;−0.470−0.074s), LHb (-0.197s;−0.396−0.002s), CA3 (-0.200s;−0.397−0.002s), V1B (-0.222s;−0.420−0.023s), V2L (-0.223s;−0.422−0.024s), Vermis (-0.213s;−0.412−0.015s), and Hemisphere (-0.272s;−0.471−0.073s). There was no difference between the T1_post_ of NS-treated and BTN-treated WT rats. *n* = 6 per group, Scheffé multiple-comparison test; *p < 0.05, **p < 0.01, ***p < 0.001. Data are presented as mean ± SEM**. (B)** Comparison of seizure latency, seizure duration, and seizure score between BTN-treated and NS-treated *Scn*1*a*^+/−^ rats at P21. BTN significantly increased seizure latency of *Scn*1*a*^+/−^ rats at P21. *n* = 6 per group, Mann–Whitney U test; *p < 0.05. Data are presented as mean ± SEM. BTN, bumetanide; NS, normal saline; P, postnatal day; M1, primary motor cortex; CI, confidence interval; M2, secondary motor cortex; CPu, caudate-putamen (striatum); LGP, lateral globus pallidus; RSGb, retrosplenial granular b cortex; DG, dentate gyrus; S1BF, primary somatosensory cortex, barrel field; Au1, primary auditory cortex; VPM, ventral posteromedial thalamic nucleus; VPL, ventral posterolateral thalamic nucleus; nRT, reticular thalamic nucleus; LHb, lateral habenular nucleus; CA3, CA3 field of the hippocampus; V1B, primary visual cortex, binocular area; V2L, secondary visual cortex, lateral area; Vermis, cerebellar vermis; Hemisphere, cerebellar hemisphere; SEM, standard error of the mean.

The decrease in T1_post_ in *Scn*1*a*^+/−^ rats occurred transiently at P19–22, and later reached the same level as wild-type rats. Thus, we speculated that the impairment of a brain developmental process that occurs at this age might contribute to this phenomenon. We focused on the maturation process of inhibitory neural circuits, which is influenced by developmental changes in intracellular chloride concentration ([Cl^−^]_i_). A decrease in [Cl^−^]_i_ promotes depolarizing to hyperpolarizing (D-H) switching and further enhances the inhibitory effect of type A gamma-aminobutyric acid (GABA_A_) receptor signaling (36, 37). On the other hand, elevated [Cl^−^]_i_ causes depolarizing GABA_A_ receptor signaling, which is observed in immature neurons primarily as a result of higher expression of the chloride importer NKCC1 (32, 36). Conversely, mature neurons express higher levels of the chloride exporter KCC2, which results in decreased [Cl^−^]_i_, leading to hyperpolarizing GABA_A_ receptor signaling (32, 36). In rodents, [Cl^−^]_i_ generally decreases by P20 (38). As a result, the D-H switch is completed by P14 (36, 37, 39), and especially by P17 in males (40, 41). Therefore, we hypothesized that the transient increase in brain activity is associated with delayed [Cl^−^]_i_ decrease. We performed a rescue experiment by administering bumetanide, an NKCC1 inhibitor (32), to *Scn*1*a*^+/−^ rats which would decrease [Cl^−^]_i_ and promote the maturation of inhibitory neural network. Bumetanide or normal saline (control) was administered intraperitoneally every day from P12–20 in wild-type rats (bumetanide-treated: *n* = 6, body weight range 31–39 g; saline-treated: *n* = 6, 31–38 g) and *Scn*1*a*^+/−^ rats (bumetanide-treated: *n* = 6, 32–40 g; saline-treated: *n* = 6, 33–37 g), and MEMRI was performed on P21 (Figure 2B). ANCOVA for T1_post_ showed no significant interaction among genotypes, ROIs, and drug administration [F_(16, 319)_ = 1.58, *p* = 0.07]. However, Scheffé multiple-comparison test with respect to the interaction showed that T1_post_ significantly increased in all ROIs, except the lateral habenular nucleus, in bumetanide-treated *Scn*1*a*^+/−^ rats compared to those in saline-treated *Scn*1*a*^+/−^ rats (*p* < 0.05; lateral habenular nucleus, *p* = 0.06), while no significant difference was observed between bumetanide- and saline-treated wild-type rats (Figure 4A). The caudate-putamen, lateral globus pallidus, retrosplenial granular b cortex, dentate gyrus, primary somatosensory cortex, reticular thalamic nucleus, and cerebellar hemisphere of *Scn*1*a*^+/−^ rats had remarkable increases in T1_post_ (*p* < 0.001). Based on these results, we speculated that the immature inhibitory neural network due to delayed decrease in [Cl^−^]_i_ is involved in the increased brain activity in *Scn*1*a*^+/−^ rats at P19–22.

### 3.5. NKCC1 inhibitor increased the threshold for heat-induced seizures in *Scn*1*a*^+/−^ rats at P21

To investigate whether bumetanide also relieves seizures in *Scn*1*a*^+/−^ rats, we examined changes in seizure characteristics in bumetanide-treated *Scn*1*a*^+/−^ rats. After the administration of bumetanide (*n* = 6, body weight range 36–39 g) or normal saline (*n* = 6, 32–41 g) to *Scn*1*a*^+/−^ rats as previously described, seizures were induced on P21 by a 45°C-hot water bath (Figure 2C). Seizure latency, seizure duration, and Racine's seizure score were compared between bumetanide- and saline-treated *Scn*1*a*^+/−^ rats. Although there was no significant difference in seizure duration (166.5 s [135.3–204.8] vs. 144.0 s [135.8–163.0], Mann–Whitney U test, U = 12.0, Z = −0.962, *p* = 0.34) or seizure score (4.0 [3.8–4.0] vs. 3.5 [2.8–4.0], Mann–Whitney U test, U = 11.5, Z = −1.251, *p* = 0.21), the seizure latency was significantly prolonged in bumetanide-treated rats (183.0 s [173.3–196.0] vs. 158.0 s [154.0–170.0], Mann–Whitney U test, U = 2.0, Z = −2.567, *p* = 0.01) (median [interquartile range]) (Figure 4B). These results indicate that bumetanide increased the seizure threshold but did not mitigate seizure severity in *Scn*1*a*^+/−^ rats.

### 3.6. NKCC1 inhibitor did not affect brain activity or heat-induced seizures in *Scn*1*a*^+/−^ rats at P27

**Figure 5:**
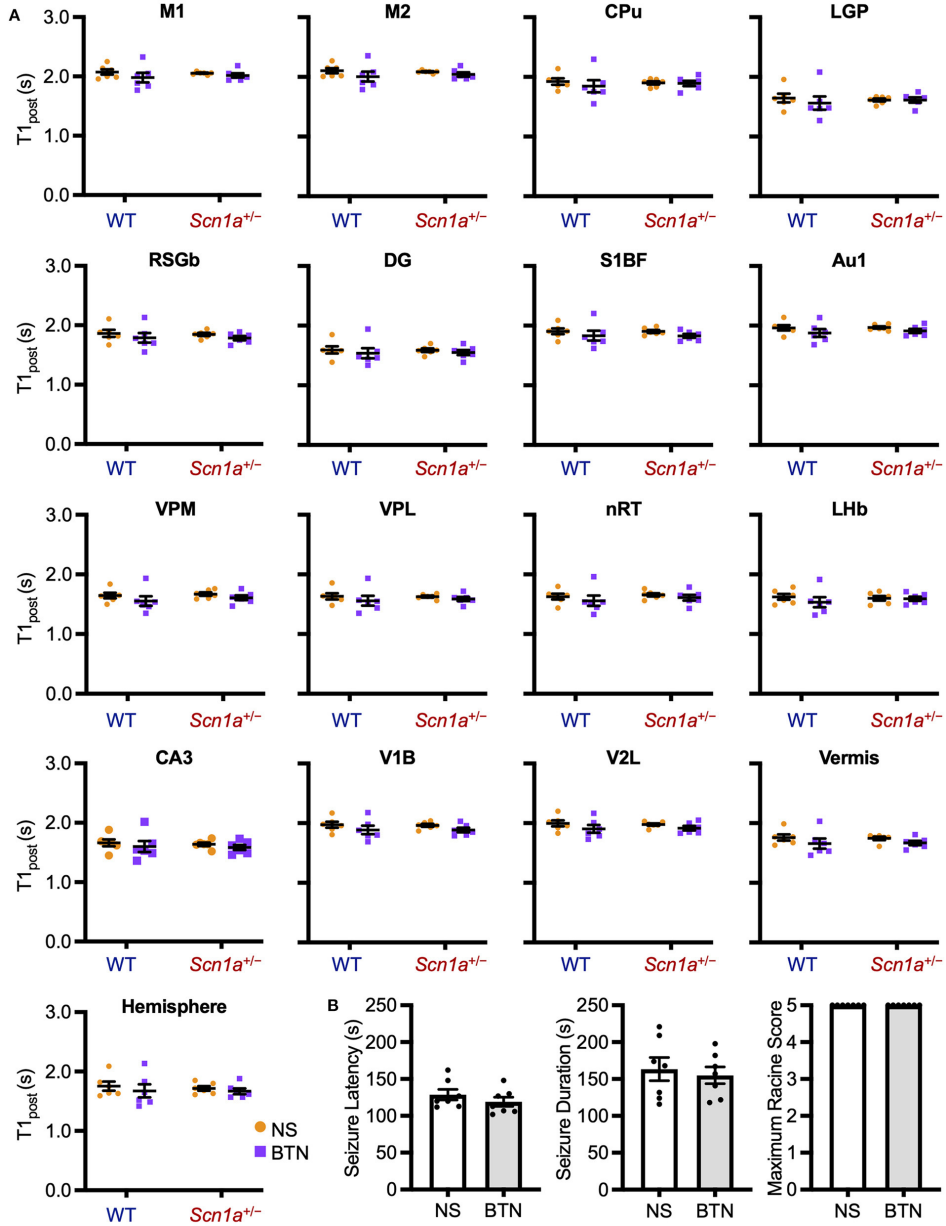
T1_post_ at P27 of wild-type and *Scn*1*a*^+/−^ rats after administration of BTN or NS. **(A)** Tukey multiple-comparison test of T1_post_ in rats at P27 after administration of BTN (orange circle) or NS (purple square). There was no significant difference in T1_post_ between BTN-treated and NS-treated *Scn*1*a*^+/−^ rats. *n* = 6 per group (Tukey multiple-comparison test). Data are presented as mean ± SEM. **(B)** Comparison of seizure latency, seizure duration, and seizure score of *Scn*1*a*^+/−^ rats at P27 between the BTN-treated and NS-treated groups. BTN did not affect the seizure characteristics at P27. *n* = 7 per group, Mann–Whitney U test. Data are presented as mean ± SEM. BTN, bumetanide; NS, normal saline; P, postnatal day; SEM, standard error of the mean.

We conducted a similar experiment in *Scn*1*a*^+/−^ rats at P27 to confirm whether the transiently observed decrease in T1_post_ meant that [Cl^−^]_i_ decrease was completed after P21. After daily bumetanide or normal saline administration from P12 to P26, we compared T1_post_ between wild-type (bumetanide-treated: *n* = 6, 44–58 g; saline-treated: *n* = 6, 44–63 g) and *Scn*1*a*^+/−^ (bumetanide-treated: *n* = 6, 62–64 g; saline-treated: *n* = 6, 60–64 g) rats using MEMRI at P27 (Figure 2B). ANCOVA for T1_post_ showed no significant interaction among genotypes, ROIs, and drug administration [F_(16, 319)_ = 0.43, *p* = 0.97]. Tukey multiple-comparison test with respect to the interaction showed no significant difference in T1_post_ between bumetanide- and saline-treated *Scn*1*a*^+/−^ rats in any ROI (Figure 5A). We also compared heat-induced seizure characteristics of bumetanide-treated (*n* = 7, 56–66 g) and saline-treated (*n* = 7, 58–69 g) *Scn*1*a*^+/−^ rats on P27 (Figure 2C). There were no significant differences in seizure latency (113.0 s [106.0–130.0] vs. 120.0 s [113.0–148.0], Mann–Whitney *U*-test, U = 16.5, Z = −1.027, *p* = 0.31), seizure duration (157.0 s [122.0–182.0] vs. 168.0 s [124.0–209.0], Mann–Whitney U test, U = 21.0, Z = −0.447, *p* = 0.66), or Racine's seizure score (5.0 [5.0–5.0] vs. 5.0 [5.0–5.0], Mann–Whitney *U*-test, U = 24.5, Z = 0.000, *p* = 1.00) (Figure 5B). These results support the hypothesis that in *Scn*1*a*^+/−^ rats, the [Cl^−^]_i_ decreased in widespread brain regions during the fourth postnatal week contributing to an overall improvement in seizure threshold.

## 4. Discussion

This study characterized the developmental changes in basal brain activity of *Scn1a* knockout rats by using MEMRI and identified a transient P19–22 hyperactivity of the brain.

Our newly generated *Scn1a* knockout rats had a milder phenotype than the *Scn*1*a*^+/−^ mice. The *Scn*1*a*^+/−^ rats showed a few spontaneous/reflex seizures, but no premature death occurred during the experimental period. Contrastingly, *Scn*1*a*^+/−^ mice show spontaneous seizures more frequently from the third postnatal week and showed premature death in about 35% of pups (18–20), mainly due to *status epilepticus* and/or malnutrition. These phenotypic differences may be due to species differences and/or epigenetic factors. Generally, rats are reported to have milder epileptic phenotypes than mice (42). However, our knockout rats had a truncating variant in *Scn1a*, a reduced brain Na_V_1.1 protein level, and heat-induced seizures were observed in all *Scn*1*a*^+/−^ rats. Additionally, all *Scn*1*a*^−/−^ rats died by the second postnatal week. These findings clearly indicate a significant involvement of the pathogenic process caused by *Scn1a* defects in our rats. The body weight gain of *Scn*1*a*^+/−^ rats was not impaired and their nutritional status was good. Thus, the MEMRI findings in *Scn*1*a*^+/−^ rats purely reflect the *Scn1a* pathology with a minimal influence of spontaneous seizures, *status epilepticus*, and malnutrition.

MEMRI revealed a significant widespread increase in the basal brain activity of *Scn*1*a*^+/−^ rats at P19–22, which corresponds to approximately 6 months of age in humans (34). This is when seizures most commonly develop in DS (1, 4, 35). Interestingly, the increase in brain activity was not significant in the fourth postnatal week. This temporal pattern of change, with stronger alterations around the onset age, has also been observed in other studies. Favero et al. (43) reported transient firing impairment of parvalbumin-positive GABAergic interneurons in *Scn*1*a*^+/−^ mice from P18 to P21, which subsided with age (43). The decrease in background EEG activity in DS mice compared to that in wild-type mice was more significant in the third postnatal week than in any other age (44). These observations indicate that the enhanced functional imbalance during this period due to impaired neuronal development associated with *Scn1a* defects possibly drives the onset and underlies frequent CSE during the early stage of DS.

The mechanisms underlying the P19–22 brain hyperactivity remain unknown. In wild-type rodents, the second to third postnatal week is the period when dynamic changes in the inhibitory network occurs. The D-H switch is mostly completed by the second postnatal week (36, 37, 39–41). The expression of Na_V_1.1 starts to increase during the second postnatal week (7, 45). A functional maturation in intrinsic electrophysiological functions of parvalbumin-positive GABAergic interneurons, such as fast spike frequencies and GABAergic synaptogenesis in fast-spiking cells, is achieved during the second to fourth postnatal week in rodents (46–48). The established mechanism of DS, impaired firing of GABAergic interneurons, especially of parvalbumin-positive cells (43), during this period, will be involved in increasing the brain activity. In this study, however, we found that bumetanide improved this hyperactivity and raised the threshold of heat-induced seizures at the third postnatal week but not at the fourth postnatal week. Thus, in the third postnatal week in *Scn*1*a*^+/−^ rats may have higher [Cl^−^]_i_ compared to wild-type rats, due to a lower expression ratio of KCC2/NKCC1, and attain the same level by the fourth postnatal week. Decrease of [Cl^−^]_i_ is a critical maturation step in GABA_A_ receptor signaling represented by D-H switch and the strength of inward Cl^−^ current. Even if the D-H switch is achieved, [Cl^−^]_i_ may still remain high as Na_V_1.1 gradually increases from the second postnatal week, resulting in a reduced inhibitory response in addition to impaired firing of GABAergic interneurons. This may lead to changes in calcium homeostasis throughout the brain in *Scn*1*a*^+/−^ rats. Subsequently, with increased Na_V_1.1 expression, [Cl^−^]_i_ may decrease and calcium homeostasis may become identical to that of wild-type. It is unclear whether catch-up is achieved in more severe phenotypes such as DS. However, the pathological changes due to Na_V_1.1-haploinsufficiency may result in delayed maturation of inhibitory neural networks in the early stage of the disease. To confirm this hypothesis, elaborate electrophysiological analysis, including postsynaptic potentials and equilibrium potentials of Cl^−^ are necessary. *In vitro* electrophysiological experiments may be significantly influenced by the effects of non-physiological brain conditions, such as the changes in neuronal properties caused by slicing or pipetting, as well as the components of the internal or extracellular solution (49), making it challenging to detect minor electrophysiological changes. Notably, a prior study using electrophysiological recordings from hippocampal CA3 pyramidal cells at P13–21 found giant depolarizing potentials and a depolarized reversal potential for GABA_A_-evoked currents in *Scn*1*b*^−/−^ mice, although this was less evident in *Scn*1*a*^+/−^ mice (50). Therefore, it is not known whether depolarizing GABA_A_ receptor signaling remains in *Scn1a*-related DS during the third postnatal week. Combined with a decreased firing capacity of GABAergic interneurons, the present results suggest that the immature and still-insufficient inhibitory response due to higher [Cl^−^]_i_ of postsynaptic neurons may be involved in the transient brain hyperactivity and seizure susceptibility at P19–22.

In our study, increased neural activity in CA3, habenular nucleus, and caudate-putamen in *Scn*1*a*^+/−^ rats at P35–38 was suggested. These findings may be related to some clinical features of DS that occur at this age, including epilepsy and nonepileptic manifestations. To elucidate this, a combination of functional neuroimaging, electrophysiology, and behavioral analysis is necessary. It would be also useful to evaluate changes in neural activity in these regions in *Scn*1*a*^+/−^ rats using MEMRI by administering several antiepileptic drugs, including carbamazepine, which is known to aggravate seizures in patients with *SCN1A* loss-of-function mutations.

The limitations of this study are as follows. First, the pathophysiology behind the transient increase in brain activity observed in the third postnatal week in *Scn*1*a*^+/−^ rats has been discussed based on the results of the bumetanide experiments and previous reports but remains a hypothetical interpretation because this has not been directly verified. As mentioned above, it is necessary to conduct high-precision electrophysiological experiments and analyze [Cl^−^]_i_, GABA reversal potentials, and the NKCC1/KCC2 expression ratio in the neurons at the single-cell level. We intend to verify this in future studies. Second, only male rats were included in this study. This was to avoid the influence of sex differences in cerebral development, including the NKCC1/KCC2 expression ratio, which has been documented (40, 41). However, future analyses in female rats are necessary for an accurate understanding of this pathology. Third, to achieve the initial goal of elucidating the pathology of DS, we generated *Scn1a* knockout rats for high-resolution MEMRI experiments. However, the phenotype of *Scn*1*a*^+/−^ rats was found to be milder than that of DS mice. Therefore, the present results showing that the differences in brain activity were no longer significant during the fourth postnatal week could have been different if MEMRI had been performed in a well-established mouse model for DS. Nevertheless, as mentioned above, the *Scn1a* knockout rats certainly reflect the pathogenesis of Na_V_1.1 haploinsufficiency and are less affected by additional factors such as status epilepticus, frequent spontaneous seizures, malnutrition, and premature death. Therefore, the *Scn1a* knockout rats are expected to be more suitable for studying the pathogenesis of *Scn1a*-related developmental encephalopathy. The MEMRI findings in *Scn*1*a*^+/−^ rats may lead to a better understanding of the pathology directly related to *Scn1a* deficiency. The *Scn*1*a*^+/−^ rats may also be used in the future to investigate various environmental factors that contribute to the formation of the DS phenotype.

In conclusion, we characterized age-dependent changes in brain activity of developing *Scn*1*a*^+/−^ rats using MEMRI that allowed high-resolution analysis at the whole-brain level *in vivo* and visualization of drug-induced changes in brain activity at each region. We believe that MEMRI can be a potential technique to uncover unrecognized aspects of various developmental and epileptic encephalopathies and can be applied to future rodent research.

## Data availability statement

The datasets presented in this study can be found in online repositories. The names of the repository/repositories and accession number(s) can be found at: figshare, https://dx.doi.org/10.6084/m9.figshare.12894698.

## Ethics statement

The animal study was reviewed and approved by the Animal Research Committee of Kyoto University and the Institutional Animal Care and Use Committee of the Jikei University School of Medicine.

## Author contributions

MT, NH, and JH contributed to the conception and design of the study. MT, NH, JH, MN, KI, SH, TK, TM, TS, and TY performed data acquisition and analysis. MT, NH, JH, MN, and HJO wrote the manuscript and prepared the figures. All authors contributed to the article and approved the submitted version.
