# Supplementary material for: Developmental changes in brain activity of heterozygous Scn1a knockout rats
Source: Front Neurol. 2023 Mar 14;14:1125089. doi: 10.3389/fneur.2023.1125089 (PMC10043303; doi:10.3389/fneur.2023.1125089)
Supplement: Supplementary file 1 [file Data_Sheet_1.docx]

Supplementary Material

Developmental changes in brain activity of heterozygous *Scn1a* knockout rats

Mayu Tahara, Norimichi Higurashi^*^, Junichi Hata, Masako Nishikawa, Ken Ito, Shinichi Hirose, Takehito Kaneko, Tomoji Mashimo, Tetsushi Sakuma, Takashi Yamamoto, Hirotaka J Okano^*^

**Correspondence:** Norimichi Higurashi: nori_@jikei.ac.jp; Hirotaka J Okano: hjokano@jikei.ac.jp

# Supplementary Figures and Video


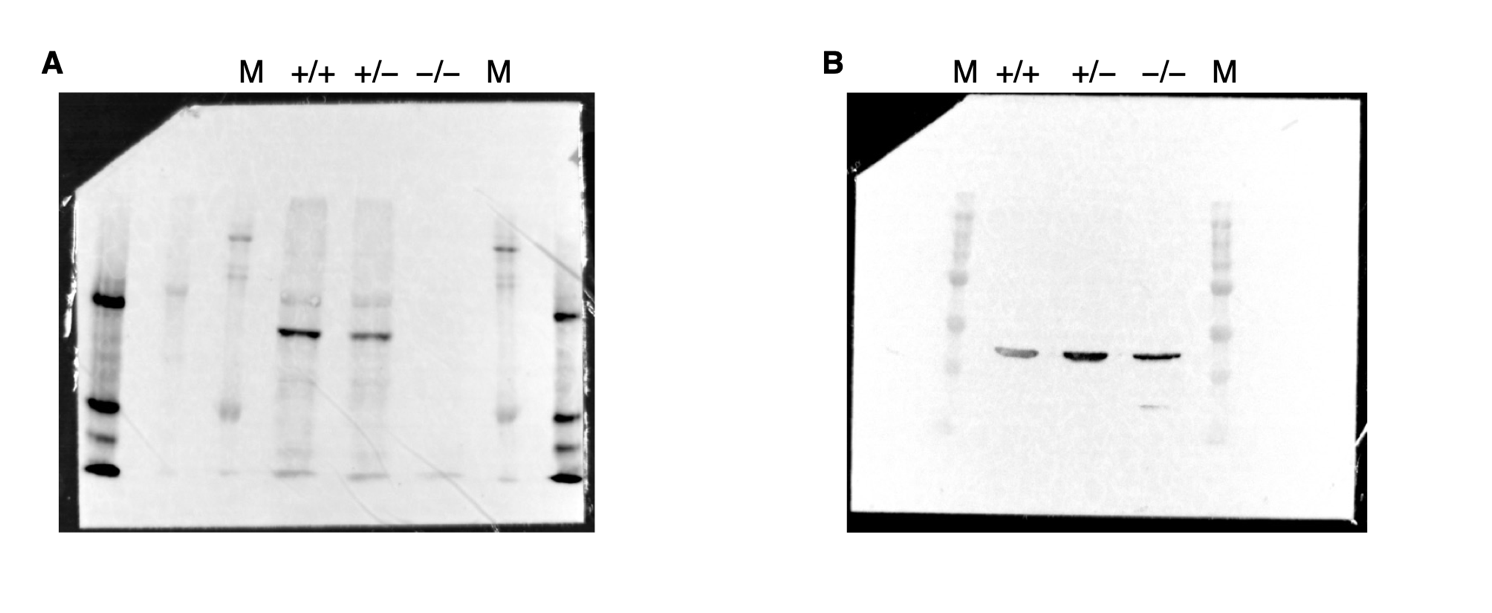


**Supplementary Figure 1.** **Full-length gels of Western blotting.**

(A) Western blotting of brain membrane proteins from wild-type (+/+), heterozygous (+/−), and homozygous (−/−) *Scn1a* rats at P13 using an anti- voltage-gated sodium channel alpha subunit 1 (Na_V_1.1) antibody. ‘M’ on both sides of the sample indicates the high molecular weight protein standards. (B) β-actin was used as the internal control. The samples were derived from the same experiment and the gels/blots were processed in parallel.


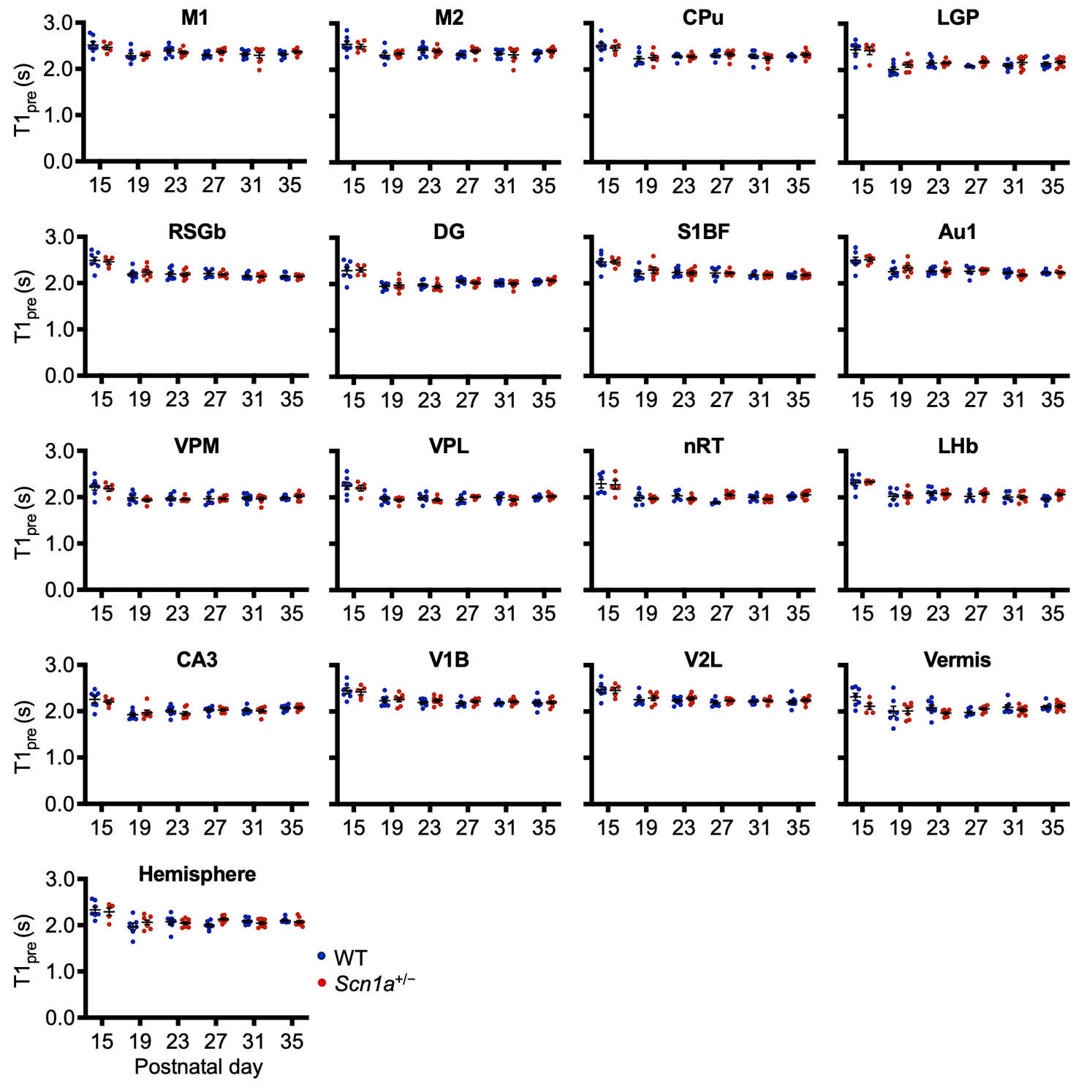


**Supplementary Figure 2. Age-dependent changes in native T1 values (T1_pre_) in each brain region of wild-type and *Scn1a*^+/^**^−^ **rats.**

T1_pre_ shows age-dependent changes in each area in WT (blue circle) and *Scn1a*^+/−^ rats (red circle). Data are presented as the mean ± standard error of the mean. WT, wild-type; M1, primary motor cortex; M2, secondary motor cortex; CPu, caudate-putamen (striatum); LGP, lateral globus pallidus; RSGb, retrosplenial granular b cortex; DG, dentate gyrus; S1BF, primary somatosensory cortex, barrel field; Au1, primary auditory cortex; VPM, ventral posteromedial thalamic nucleus; VPL, ventral posterolateral thalamic nucleus; nRT, reticular thalamic nucleus; LHb, lateral habenular nucleus; CA3, CA3 field of the hippocampus; V1B, primary visual cortex, binocular area; V2L, secondary visual cortex, lateral area; Vermis, cerebellar vermis; Hemisphere, cerebellar hemisphere.

Supplementary Video 1 (separate file). Seizure induced by hot water in an *Scn1a*^+/-^ rat.

Severity of the seizure is equivalent to Racine score 3 to 5.
